# Long-term neuropsychological outcomes in children with febrile infection-related epilepsy syndrome (FIRES) treated with anakinra

**DOI:** 10.3389/fneur.2023.1100551

**Published:** 2023-03-08

**Authors:** Anima Shrestha, E. Lynne Wood, Gretchen Berrios-Siervo, Coral M. Stredny, Katrina Boyer, Clemente Vega, Srishti Nangia, Eyal Muscal, Krista Eschbach

**Affiliations:** ^1^University of Colorado School of Medicine, Aurora, CO, United States; ^2^Department of Pediatrics, Section of Neurology, University of Colorado School of Medicine, Children's Hospital Colorado, Aurora, CO, United States; ^3^Department of Pediatrics, University of Colorado School of Medicine, Children's Hospital Colorado, Aurora, CO, United States; ^4^Division of Epilepsy and Clinical Neurophysiology, Department of Neurology, Boston Children's Hospital, Boston, MA, United States; ^5^Program in Neuroimmunology, Department of Neurology, Boston Children's Hospital, Boston, MA, United States; ^6^Department of Child Neurology, Weill Cornell Medicine, New York-Presbyterian Hospital, New York, NY, United States; ^7^Department of Pediatrics and Child Neurology (Co-appointment), Baylor College of Medicine, Houston, TX, United States

**Keywords:** new-onset refractory status epilepticus, NORSE, febrile infection-related epilepsy syndrome, FIRES, neuropsychological outcomes, cognitive outcomes, anakinra

## Abstract

**Background:**

Febrile-infection related epilepsy syndrome (FIRES) is a rare epilepsy syndrome in which a previously healthy individual develops refractory status epilepticus in the setting of a preceding febrile illness. There are limited data regarding detailed long-term outcomes. This study aims to describe the long-term neuropsychological outcomes in a series of pediatric patients with FIRES.

**Methods:**

This is a retrospective multi-center case series of pediatric patients with a diagnosis of FIRES treated acutely with anakinra who had neuropsychological testing at least 12 months after status epilepticus onset. Each patient underwent comprehensive neuropsychological evaluation as part of routine clinical care. Additional data collection included the acute seizure presentation, medication exposures, and outcomes.

**Results:**

There were six patients identified with a median age of 11.08 years (IQR: 8.19–11.23) at status epilepticus onset. Anakinra initiation was a median of 11 days (IQR: 9.25–13.50) after hospital admission. All patients had ongoing seizures and none of the patients returned to baseline cognitive function with a median follow-up of 40 months (IQR 35–51). Of the five patients with serial full-scale IQ testing, three demonstrated a decline in scores over time. Testing results revealed a diffuse pattern of deficits across domains and all patients required special education and/or accommodations for academic learning.

**Conclusions:**

Despite treatment with anakinra, neuropsychological outcomes in this series of pediatric patients with FIRES demonstrated ongoing diffuse neurocognitive impairment. Future research will need to explore the predictors of long-term neurocognitive outcomes in patients with FIRES and to evaluate if acute treatment interventions improve these outcomes.

## Introduction

New-onset refractory status epilepticus (NORSE) is a clinical presentation in which previously healthy individuals develop refractory status epilepticus without a clear structural, toxic, or metabolic cause. Febrile infection-related epilepsy syndrome (FIRES) is a subset of NORSE that is preceded by febrile illness 24 hours to 2 weeks prior to the onset of status-epilepticus (1). Children with FIRES will present after a febrile illness with new seizures that rapidly progress to refractory or super refractory status epilepticus that can last for several weeks despite treatment with at least two intravenous antiseizure medications and continuous infusions (2, 3). Limited published literature to date suggests that of patients who survive the acute phase, nearly all have ongoing drug-resistant epilepsy with the majority not returning to prior baseline function (4, 5).

No etiology is identified in the majority of FIRES cases, but there is growing evidence that there may be an immune-mediated process following an initial infection (6). A preceding febrile illness can trigger intrathecal overproduction and release of proinflammatory cytokines, which activates mechanisms of innate immunity in the central nervous system (7). This immune activation increases neuronal excitability, leading to epileptogenesis (8). Status epilepticus itself can trigger proinflammatory processes and neuroinflammation which further promotes neuronal hyperexcitability and triggers ongoing seizures (9, 10). This hypothesized immune activation has led to the use of immune therapies such as steroids, intravenous immunoglobulin (IVIG), and plasma exchange in the treatment of FIRES, though typically with low response rates (11). One particular proinflammatory cytokine, interleukin-1 beta (IL-1β) has been implicated in experimental models of status epilepticus (12). Anakinra is an interleukin-1 receptor antagonist (IL-1Ra) used in FIRES that has been shown to reduce seizures in the acute phase (5, 6, 13) and is recommended for consideration in the treatment of patients with FIRES (14, 15).

Published data regarding the long-term neurocognitive outcomes in patients with FIRES is limited. Despite reports of acute benefit with anakinra, there are no prior studies describing long-term cognitive outcomes in this population. This study aims to describe the long-term neuropsychological and seizure outcomes in a series of pediatric patients with FIRES treated with anakinra.

## Materials and methods

This is a multicenter retrospective case series of six patients with a diagnosis of FIRES treated with anakinra. Treating physicians identified patients with FIRES onset between December 2015 and December 2019. Inclusion criteria included patients < 18 years old with a diagnosis of FIRES treated with anakinra in the acute hospital admission with available neuropsychological testing at least one year after refractory status epilepticus onset. Study data were collected and managed using REDCap electronic data capture tools hosted at University of Colorado School of Medicine (16). Data collected included past medical and family history, lab and imaging results during the acute hospital course, as well as seizure frequency, antiseizure medications and other treatments (i.e., neuro-modulatory therapy) during the acute hospital course, and at each follow-up neuropsychological testing time point. Neuropsychological testing was completed as part of routine clinical care across three academic medical centers. Testing measures varied by institution, age of the patient at the time of assessment, and ability to complete measures. For analysis, tests were grouped into neuropsychological domains, and scores described between and within categories. The Colorado multiple institutional review board (COMIRB) approved this study.

Descriptive data analysis included frequencies and percentages for categorical variables. Continuous variables are reported in median and interquartile ranges. A pre-determined sample size was not calculated due to descriptive nature of study and small number of available patients.

## Results

There were six patients identified with a median age of 11.08 years (Interquartile range (IQR) 8.19–11.23) at status epilepticus onset. All patients met the diagnostic criteria for FIRES with a preceding fever starting at least 24 hours prior to status epilepticus onset. Initial brain MRI was normal in three patients and non-specific inflammation such as T2/FLAIR hyperintensities occurred in three patients. Completion of genetic testing occurred in five patients and was negative or non-diagnostic in all these children. All patients had autoimmune antibody testing completed in cerebrospinal fluid (CSF), as well as in serum in five patients. This was negative in all patients except for one with a low-titer serum glutamic acid decarboxylase 65 (GAD65) (0.09 nmol/L; normal < 0.02 nmol/L) only after treatment with IVIg. Four children had CSF cytokine testing, which were all elevated (Table 1). During the acute phase, all patients received treatment with pentobarbital and obtained burst-suppression on EEG. The median duration of burst suppression was 120 hours (IQR 63–348). All patients received treatment with intravenous immunoglobulins (IVIg) and five patients received corticosteroids. These treatments were prior to anakinra in four patients and concurrent with anakinra in two. The median time to initiation of anakinra was 11.0 days (IQR 9.25–13.50). One patient received treatment with tocilizumab following anakinra; this case was previously published and is included in this series to report additional long-term outcome data (17). Five patients received treatment with phenobarbital during the acute period. There were ongoing seizures at last follow-up for all patients with variable frequency ranging from multiple per day to monthly. All patients continued to take a median of 3.5 (IQR 2–5) daily antiseizure medications at the time of their last neuropsychological evaluation. Two patients also received treatment with neuromodulation (vagus nerve stimulation or responsive neurostimulation). Brain MRI results in the long-term follow-up phase revealed a combination of hippocampal volume loss, including mesial temporal sclerosis, and / or diffuse cerebral or cerebellar volume loss (Table 1). Additional information regarding specific cytokine testing results, duration of treatment with anakinra, antiseizure medication exposure, and seizure outcomes are available in Table 1.

**Table 1:** Patient characteristics and medical history.

**Patient **	**Age at onset (years) **	**ICU length of stay (days) **	**CSF cytokineelevations**	**Time in burst suppression (hours) **	**# ASM attempted during inpatient admission **	**Immune-modulatory treatment besides anakinra **	**Time to anakinra start (days) **	**Time on anakinra (days) **	**Seizure burden prior to anakinra **	**Seizure burden at hospital discharge **	**Seizure burden atlast evaluation**	**ASM at last cognitive evaluation**	**Rescue medication frequency at last cognitive evaluation**	**Follow-up MRI results (months since onset) **
**1**	11	40	IL-1B, IL-4, IL-5, IL-6, IL-8, IL-10, IFN-γ	48	9	Corticosteroids IVIg Plasmapheresis	14	83	100 per day	1 per month	2.5 per month	Clobazam Lacosamide Phenobarbital	1 every 6 months	Low hippocampal volume with concern for unilateral mesial temporal sclerosis (51 months)
2	11	16	IL-1, IL-4, IL-6, IL-8, IL-10	72	7	Corticosteroids IVIg	16	46 – later restarted	18 per day	2 per month	1 per day	Clobazam Valproic acid	Monthly	Progression of cerebral and cerebellar volume loss, unilateral hippocampal volume loss and asymmetric T2 signal (40 months)
3	11	31	Not checked	60	9	Corticosteroids IVIg	19	Remains on anakinra	52 per day	1 per month	1 per day	Lacosamide Brivaracetam VNS Bilateral hippocampal RNS	Infrequent	Slight increase in moderate to marked cerebellar volume loss (81 months)
4	11	55	Not checked	408	10	IVIg	9	26	22 per day	1-2 per week	1 per week	Clobazam Lacosamide Phenobarbital Levetiracetam Lorazepam	3 per month	Right greater than left diffuse atrophy; left greater than unilateral hippocampal volume loss (58 months)
5	7	60	IL8, IL5, IP-10 (CXCL10)	720	8	Corticosteroids IVIg	50	Remains on anakinra	4 per week	0	Daily	Clobazam Lacosamide Levetiracetam Perampanel Decadron VNS	None	Stable mild generalized volume loss, unchanged likely mesial temporal sclerosis that is asymmetric (50 months)
6	6	53	IL-2, IL-4, IL-6, IL-8, IL-10	168	9	Corticosteroids IVIg	5	15	42 per day	1–2 per week	2 per day	Phenobarbital Levetiracetam Epidiolex Rufinamide Oxcarbazepine	2–3 per month	Right mesial temporal sclerosis with mild generalized atrophy (27 months)

IL, interleukin; IFN-γ, interferon gamma; ICU, intensive care unit; CSF, cerebrospinal fluid; ASM, antiseizure medications; VNS, vagus nerve stimulation; RNS, responsive neurostimulation.

**Figure 1:**
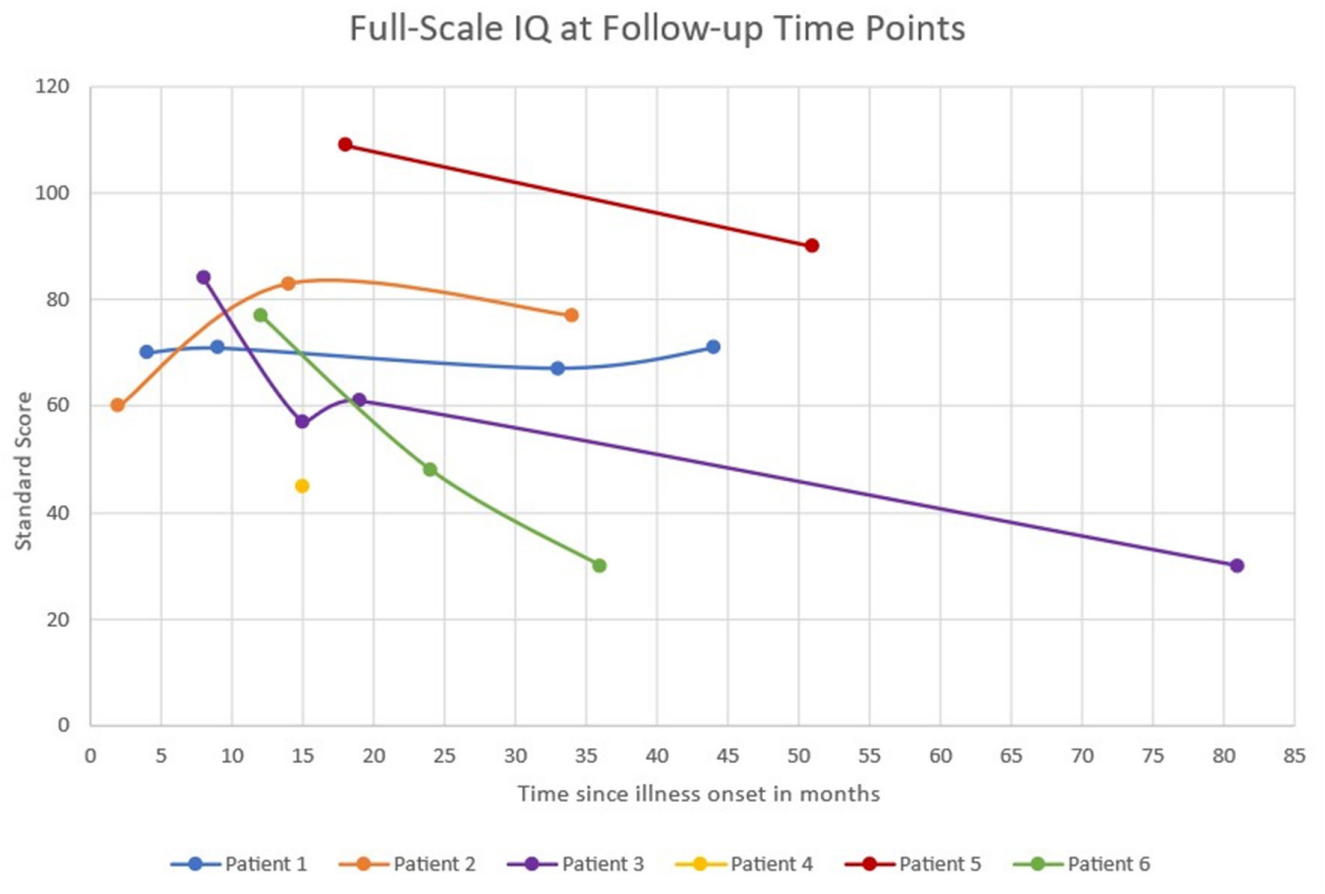
Full-scale IQ scores at serial follow-up time points. Full-scale IQ score categories: Average 90–109, Low average 80–89, Borderline 70–79, Extremely low <69.

Patients had serial neuropsychological testing repeated between 1 and 4 times after status epilepticus onset with a median follow-up of 40 months (IQR 35–51 months). The testing measures completed for each patient during follow-up are available in Supplementary Table 1. None of the six patients returned to baseline functioning after onset of FIRES, according to physician report and pediatric cerebral performance category (PCPC). PCPC was normal for all patients prior to FIRES onset with all patients demonstrating decline in function to a score of moderate to severe disability at last follow-up (Table 2). There was one patient (patient 2) who had improvement in PCPC score over time with a change from the category of severe disability to moderate disability. This patient also had an improvement in full-scale IQ score from initial testing (Figure 1). Full-scale IQ testing for one patient (patient 5) demonstrated average IQ scores, although there was a decline in scores between follow-up time points. The remainder of patients demonstrated IQ scores that were between the ranges of low average to extremely low. Of the five patients with serial IQ testing, three patients demonstrated a decline in full-scale IQ scores over the follow-up period.

**Table 2:** Patient outcome measures according to physician report.

**Patient #**	**PCPC at admission**	**PCPC at discharge**	**PCPC at last follow-up**	**Return to neurocognitive baseline**	**Ambulation**	**Return of speech**	**Return to school**	**Additional academic supports**
1	1	3	3	No	Independent	Yes	Yes	Medical day program followed by IEP, special education classes
2	1	4	3	No	Independent	Yes	Yes	Medical day treatment program
3	1	4	4	No	Independent	Yes	Yes	Homebound tutor
4	1	4	4	No	Independent	Yes	Yes	Special education classes, IEP
5	1	3	3	No	Independent	Yes	No	Special education classes
6	1	3	3	No	Independent	Yes	Yes	1:1 paraprofessional or tutor

PCPC, Pediatric Cerebral Performance Category; IEP, individualized education plan.

**Figure 2:**
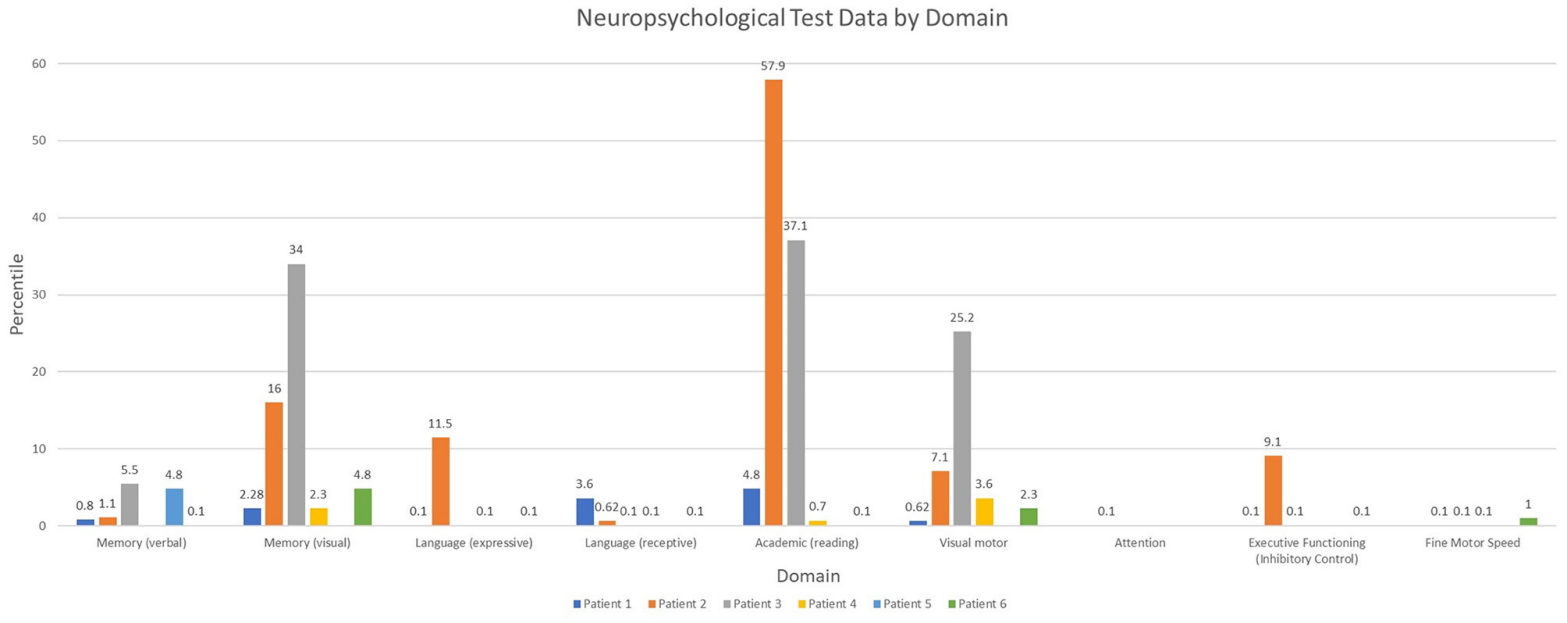
Neuropsychological test data by domain at the time of last assessment.

Overall, patients demonstrated a diffuse pattern of deficits across domains at the last follow-up time point (Figure 2). Deficits included verbal and non-verbal reasoning and visual spatial abilities, as well as memory deficits. Language weaknesses encompassed both receptive and expressive language impairment. Additionally, visual motor integration, fine motor speed and dexterity were each below age expectations, and weaknesses occurred across varying aspects of attention and executive functioning. Reading skills ranged from extremely low to average. Longitudinal testing demonstrated preservation of these skills over time, without evidence for decline or loss of skills. While longitudinal data was not available for other areas, there was a slight trend for lower math calculation skills when compared to reading. Spelling was more variable.

Parent, and in some cases, teacher report questionnaires (ABAS-3, BASC-3, and Vineland-3) were suggestive of concerns for symptoms of executive functioning deficits, psychosocial difficulties and overall adaptive functioning weaknesses in at least two patients. Parent or teacher BASC-3 questionnaires were completed in three children and suggestive of clinically significant hyperactivity. In two children, there was also indication of internalizing symptoms, including increased anxiety, somatization, or depression. At last follow-up, all patients continued to present with neuropsychological impairments and required special education services/interventions and/or accommodations for academic learning (Table 2).

## Discussion

In this series of pediatric patients with FIRES treated acutely with anakinra, neuropsychological testing does not suggest a pattern of specific neurocognitive deficits, but rather a global decline in functioning. There is evidence that some patients continue to have further neurocognitive decline over time, although some patients demonstrate stable neuropsychological scores or improvement. All patients required educational support and interventions during the long-term follow-up period.

These findings are concordant with data supporting that super-refractory status epilepticus causes worse neurological outcomes and lower rates of return to baseline cognitive function than non-super-refractory status epilepticus (18). One study reported that 58% of patients with NORSE had neurocognitive deficits, of which 30% were severe (19). This is consistent with the current case series in which all patients demonstrate neurocognitive deficits including two patients classified as severe based on PCPC category. All patients in this series had return of speech and independent ambulation although no patients returned to their neurocognitive baseline, and all had some degree of disability at follow-up.

In general, patients had full-scale IQs below normal, and three patients showed a decline in full-scale IQ during period of long-term follow-up. This aligns with a previous study in which all patients with devasting epileptic encephalopathy in school-aged children (DESC) with prolonged status epilepticus had IQs below normal at follow-up, and serial follow-up neurocognitive testing showed progressive decline of intellectual functioning (19). At follow-up in another series of children with FIRES only 18% of survivors returned to normal function at last follow-up, although this classification also included the presence of learning disabilities (4). Poor cognitive outcomes were associated with younger age at FIRES onset and longer duration of induced burst suppression coma (4). The current case series reflects this with the youngest patients at FIRES onset exhibiting the second and third largest declines in IQ over the follow-up period with one of these patients also spending the longest time in burst suppression. However, this case series is not powered to statistically make comparisons between exposures. Three patients demonstrated ongoing decline in serial full-scale IQ over time. This decline may be multifactorial and related not only to initial FIRES presentation with refractory status epilepticus but also partly due to ongoing seizure activity in the chronic phase, medication effect, and factors related to the underlying etiology and pathophysiology of FIRES that need further exploration. However, it is also important to note the variability in serial full-scale IQ testing in this case series with one patient improving over time. This suggests that recovery of neurocognitive function can occur for some patients and highlights the importance of rehabilitation programs to maximize this potential.

When further evaluating domains of neuropsychological function this case series identified concern in all domains including verbal and non-verbal memory and visual spatial skills, as well as language weaknesses with very low receptive and expressive language scores and reduced fine motor speed and dexterity. Concordant with the present findings, neuropsychological testing in children with convulsive status epilepticus show decreased memory scores compared to a healthy control group (20). Though this study did not compare FIRES patients to a control group, all patients in this study had a verbal and visual memory score at last follow-up that fell in the bottom two quartiles. Additionally, there are prior reports of FIRES patients presenting with impairments in word finding, fluency, knowledge of words, and semantic comprehension (4, 19, 21).

Attention and executive functioning are also greatly impaired in patients with a diagnosis of FIRES. Executive function deficits are also seen in patients with drug-resistant frontal and temporal epilepsy and reduced impulse-inhibition in patients with temporal lobe epilepsy (22). These findings and those in the present case series may be in part due to ongoing drug-resistant epilepsy, localization of seizure onset, and cerebral dysfunction in the chronic phase of FIRES with multiple daily antiseizure medications.

Academic achievement was among the best performing domains for patients in this case series. This aligns with reports of higher scores for specific academic skills (such as arithmetic, spelling, reading recognition, and reading comprehension) relative to academic performance in patients with traumatic brain injury. Cognitive and behavioral deficits that impact performance may mediate this discrepancy (23).

Social and emotional functioning results were variable on parent report suggesting concerns for internalizing (attention, anxiety, depression) or externalizing (hyperactivity, aggression, conduct problems) for some patients although not all. In a prior study of children with DESC, all patients presented with emotional and behavioral disorders that included fits of anger, aggressiveness, agitation, apathy, and withdrawal behaviors (19). Another study of patients with convulsive status epilepticus found that approximately one-third of patients scored above the clinical cut-off on at least one behavioral scale (24). It is therefore reasonable to consider that these behavioral impairments may influence reports of academic performance in relation to academic skills. It is important to evaluate and address behavior and social-emotional functioning in the clinical care of patient with FIRES.

Most patients in this case series returned to some form of education but all had special education needs. This is reflected in the literature with prior reports of FIRES needing special education classes (19) and half of children with convulsive status epilepticus having special educational needs (21).

Overall, the described changes in cognitive function have multiple proposed explanations including the location of seizures, initial refractory status epilepticus, ongoing drug-resistant epilepsy, as well as antiseizure medication exposure in the acute and chronic phase of FIRES treatment. Additional causative considerations include the impact of the underlying pathophysiology of FIRES and status epilepticus. Seizures in children with FIRES often have a focal onset in both temporal-perisylvian areas of the brain, then spread to the frontal lobes (9, 25). PET scans show hypometabolism in the temporoparietal and orbitofrontal cortices (25). Damage in these areas is consistent with cognitive deficits found in FIRES, most commonly language, memory, behavior, and frontal lobe function. Follow-up brain imaging in this case series demonstrates diffuse atrophy and mesial temporal sclerosis or hippocampal volume consistent with prior reports in the chronic phase (26). These findings may additively contribute to cognitive impairments and future research evaluating serial magnetic resonance imaging (MRI) of the brain with long-term neurocognitive outcomes is warranted.

Antiseizure medication themselves can also contribute to cognitive and behavioral deficits. During the acute and chronic phases of FIRES patients receive treatment with multiple antiseizure medications as shown in this cohort with all patients receiving treatment with at least two antiseizure medications at the time of their last follow-up testing. Specifically high dose phenobarbital has been proposed in the acute treatment of FIRES (14) and in our cohort five patients received this treatment acutely with two patients remaining on phenobarbital at the last follow-up neuropsychological testing visit. Phenobarbital has been associated with an IQ that is on average 8.4 points lower than placebo in children with febrile seizures, as well as reduced performance on IQ when compared to valproic acid (27–29). There are additional antiseizure medications that can also be associated with cognitive and behavioral changes, such as topiramate or levetiracetam (29, 30). Overall, the cognitive and behavioral side effects of antiseizure medications may explain *some* of the cognitive deficits seen in this study population, although antiseizure medications are unlikely to account for the full extent of decline compared to baseline function prior to onset of FIRES.

One limitation of this study is small sample size, as FIRES is a rare pediatric condition, and only patients treated with anakinra were included. Additionally, because this was a multicenter, retrospective study, neuropsychological testing could not be standardized across all sites and patients, so patients received various neuropsychological tests at various timepoints. In some cases, alternative testing procedures were utilized as a result of the patient's cognitive limitations (e.g., use of WNV instead of WISC-V or WASI-II in a patient that presented with severe language deficits). While there was often no direct comparison of scores between or within patients, percentiles could be compared within the same category of neuropsychological tests. This allowed for an adequate understanding of patients' neurocognitive functioning at last follow-up compared to their peers. However, standardized serial monitoring of neuropsychological outcomes would be beneficial as part of routine follow-up care for patients with FIRES.

Future prospective studies of patients with FIRES in which standardized neuropsychological assessments are completed at set follow-up points would allow for a more direct analysis of change in neurocognitive function over time. This should be correlated with detailed neuroimaging and electrographic data to better understand these outcomes over time. It would also be helpful to include a comparative control group of patients not treated with anakinra to better determine any potential benefits of anakinra on neuropsychological outcomes. Ongoing multicenter collaboration and family engagement are encouraged to meet these objectives.

## Conclusions

In summary, long-term follow-up of FIRES patients treated with anakinra demonstrates significant neurocognitive impairment across all neuropsychological testing domains with variable stability of scores over time. This reflects and builds upon neuropsychological data available in the literature. Future research needs to better understand the predictors of long-term neurocognitive outcomes and influence of acute treatment interventions. There is a need for standardized long-term serial neuropsychological assessments for all patients with FIRES and NORSE. It is recommended that this be incorporated into a multidisciplinary approach including not only neurologists and epilepsy specialists, as well as rehabilitation, neuropsychology, and mental health team members to support maximal neurocognitive recovery and support.

## Data availability statement

The original contributions presented in the study are included in the article/Supplementary material, further inquiries can be directed to the corresponding author.

## Ethics statement

The studies involving human participants were reviewed and approved by Colorado Multiple Institutional Review Board (COMIRB). Written informed consent from the participants' legal guardian/next of kin was not required to participate in this study in accordance with the national legislation and the institutional requirements.

## Author contributions

EW, GB-S, EM, and KE contributed to the conception and design of the study. EW and KE developed the database. CS, KB, CV, SN, EW, and KE completed data collection. AS, EW, GB-S, and KE performed data review and analysis. EW and AS wrote the initial drafts of the manuscript. All authors contributed to manuscript revision, read, and approved the submitted version.
