# Supplementary material for: Long-term neuropsychological outcomes in children with febrile infection-related epilepsy syndrome (FIRES) treated with anakinra
Source: Front Neurol. 2023 Mar 8;14:1100551. doi: 10.3389/fneur.2023.1100551 (PMC10030614; doi:10.3389/fneur.2023.1100551)
Supplement: Supplementary file 1 [file Table_1.docx]

Supplementary Table 1: Neuropsychology testing administered for each patient

| Patient # | IQ | Memory | Language | Visual-Motor / Visual-Perceptual skills | Attention and Executive Functioning | Motor / Sensory | Academic Achievement | Social and Emotional Functioning / Adaptive Skills |
| --- | --- | --- | --- | --- | --- | --- | --- | --- |
| 1 | WISC-V  WASI-II | CVLT-C  ChAMP | BNT-2  MAE-3 | VMI-6  Hooper | DKEFS  WCST |  | WIAT-III | ABAS-3 |
| 2 | WISC-V  WASI-II | CVLT-C  WRAML-2  ChAMP  ROCF | BNT-2  CELF-5  MAE-3 | VMI-6 | CPT-3  DKEFS  NEPSY-II  TOL-2  WCST  BRIEF-2  NICHQ Vanderbilt | Grooved Pegboard Sensory Perceptual Exam | WIAT-III  WIAT-IV | BASC-3  RCADS  ABAS-3 |
| 3 | WISC-V  WASI-II | CVLT-C  ROCF |  | VMI-6 | DKEFS | Grooved Pegboard | WIAT-III | ABAS-3 |
| 4 | WNV | ROCF  BVMT-R  CMS | BNT-2  CELF-5  EOWPVT-4  ROWPVT-4 | VMI-6 | CPT-3  DKEFS  NEPSY-II  BRIEF-2 | Grooved Pegboard | WIAT-III | BASC-3, parent + teacher report  Vineland 3 |
| 5 | WISC-V  WASI-II | CVLT-C |  |  | DKEFS |  |  |  |
| 6 | WPPSI-III  DAS-II | CMS | CELF-5  PPVT-5  EVT-3  NEPSY-II | VMI-6 | NEPSY-II  BRIEF-2 | NEPSY-II | WJ-IV  WIAT-IV | BASC-3, parent + teacher report  ABAS-3 |

WISC-V: Wechsler Intelligence Scale for Children, Fifth Edition; WASI-II: Wechsler Abbreviated Scale of Intelligence, Second Edition; WNV: Wechsler Nonverbal Scales of Ability; WPPSI-III: Wechsler Preschool and Primary Scale of Intelligence, Third Edition; DAS-II: Differential Ability Scales, Second Edition; CVLT-C: California Verbal Learning Test, Children's Version; ChAMP: Children and Adolescent Memory Profile; WRAML-2: Wide Range Assessment of Memory and Learning, Second Edition; ROCF: Rey-Osterrieth Complex Figure; BVMT-R: Brief Visual Memory Test, Revised; CMS: Children’s Memory Scale; BNT-2: Boston Naming Test, Second Edition; MAE-3: Multilingual Aphasia Examination- Third Edition; CELF-5: Clinical Evaluation of Language Fundamentals, Fifth Edition; EOWPVT-4: Expressive One-word Picture Vocabulary Test, Fourth Edition; ROWPVT-4: Receptive One-word Picture Vocabulary Test, Fourth Edition; PPVT-5: Peabody Picture Vocabulary Test, Fifth Edition; EVT-3: Expressive Vocabulary Test, Third Edition; NEPSY-II: A Developmental Neuropsychological Assessment, Second Edition; VMI-6: Beery-Buktenica Developmental Test of Visual-Motor Integration, Sixth Edition; DKEFS: Delis Kaplan Executive Function System; WCST: Wisconsin Card Sorting Test; CPT-3: Conners' Continuous Performance Test III; TOL-2: Tower of London-Drexel, Second Edition; BRIEF-2: Behavior Rating Inventory of Executive Function, Second Edition (BRIEF-2), Parent report; NICHQ Vanderbilt: National Institute for Children’s Health Quality Vanderbilt Assessment Questionnaire, Parent report; WIAT-III: Weschler Individual Achievement Test, Third Edition; WIAT-IV: Weschler Individual Achievement Test, Fourth Edition; WJ-IV: Woodcock-Johnson, Fourth Edition; ABAS-3: Adaptive Behavior Assessment System, Third Edition; BASC-3: Behavior Assessment System for Children, Third Edition; RCADS: Revised Children’s Anxiety and Depression Scale, Parent Report; Vineland-3: Vineland Adaptive Behavior Scales, Third Edition
